# Neutron reflectivity measurement of protein A–antibody complex at the solid-liquid interface

**DOI:** 10.1016/j.chroma.2017.03.084

**Published:** 2017-05-26

**Authors:** Alice R. Mazzer, Luke A. Clifton, Tatiana Perevozchikova, Paul D. Butler, Christopher J. Roberts, Daniel G. Bracewell

**Affiliations:** a Dept. Biochemical Engineering, University College London, Gower Street, London, WC1E 6BT, UK; b ISIS, Rutherford Appleton Laboratory, Harwell Science and Innovation Campus, Didcot, Oxon, OX11 0QX, UK; c Department of Chemical and Biomolecular Engineering, University of Delaware, Newark, DE, 19716, USA; d National Institute of Standards and Technology, 100 Bureau Drive, Bldg. 235, Gaithersburg, MD 20899-8562, USA

**Keywords:** Protein A, Neutron reflectivity, Antibody, Chromatography, Adsorption, Interface

## Abstract

Chromatography is a ubiquitous unit operation in the purification of biopharmaceuticals yet few studies have addressed the biophysical characterisation of proteins at the solution-resin interface. Chromatography and other adsorption and desorption processes have been shown to induce protein aggregation which is undesirable in biopharmaceutical products. In order to advance understanding of how adsorption processes might impact protein stability, neutron reflectivity was used to characterise the structure of adsorbed immunoglobulin G (IgG) on model surfaces. In the first model system, IgG was adsorbed directly to silica and demonstrated a side-on orientation with high surface contact. A maximum dimension of 60 Å in the surface normal direction and high density surface coverage were observed under pH 4.1 conditions. In chromatography buffers, pH was found to influence IgG packing density and orientation at the solid-liquid interface. In the second model system, which was designed to mimic an affinity chromatography surface, protein A was attached to a silica surface to produce a configuration representative of a porous glass chromatography resin. Interfacial structure was probed during sequential stages from ligand attachment, through to IgG binding and elution. Adsorbed IgG structures extended up to 250 Å away from the surface and showed dependence on surface blocking strategies. The data was suggestive of two IgG molecules bound to protein A with a somewhat skewed orientation and close proximity to the silica surface. The findings provide insight into the orientation of adsorbed antibody structures under conditions encountered during chromatographic separations.

## Highlights

• The orientation of IgG4 adsorbed at the solid-liquid interface was probed.
• A chromatography resin was mimicked by attaching protein A to a silica surface.
• Neutron reflectivity was used to measure protein A and adsorbed IgG structures.
• Protein A-modified silica was blocked with either BSA or PEG before IgG adsorption.
• Adsorbed IgG extended up to 230 Å from the surface, depending on blocking strategy.

## 1 Introduction

Sorption events at the solid-liquid interface in preparative chromatography are critical not only to separation but also product stability; in commercial separations maximising binding capacity and maintaining product quality and yield are critical. The highly specific IgG-protein A interaction is widely exploited for purification of monoclonal antibodies. In previous work we showed that IgG4 eluted from a protein A column aggregated more rapidly at low pH than that which had not undergone an adsorption-desorption event [1]. Other recently published work supports the theory that IgG eluted from protein A is more susceptible to aggregation induced by a secondary stress [2]. In two related studies, it was shown that IgG1 undergoes significant changes in average hydrodynamic radius and secondary structure content during elution in protein A chromatography [2], [3]. Gagnon and Nian [3] suggested that IgG1 size-reduction was influenced largely by protein concentration, a theory supported by other studies [4]; changes in secondary structure were proposed to be the result of a “dual site” interaction of IgG with protein A, whereby each IgG molecule bound two protein A molecules [3].

Previous work has necessarily relied on indirect methods to infer details of adsorbed proteins. An improved understanding of the adsorption process would benefit from direct measurement of the adsorbed protein species. One method of measuring adsorbed biological structures is neutron reflection. Neutron reflectometry provides a means to obtain *in situ*, meso-scale structural information on biological films as a function of solution conditions and different surface environments, without causing damage to samples. Neutron beams interact with atomic nuclei, allowing them to pass long distances (centimetres) through certain materials without being scattered or adsorbed. This penetrating power makes neutrons particularly useful for studying layered structures and buried interfaces using reflectometry methods [5], [6], [7].

A number of studies have used neutron reflection methods to characterise the structure of proteins adsorbed directly to silica, and determine the influence of mobile phase composition and protein concentration on adsorbed protein structures [8], [9], [10], [11], [12], [13]. Most recently, Perevozchikova et al. [11] used reflectivity to characterise an IgG1 adsorbed to silica in sodium citrate at pH 4.5 and pH 6.2 so as to assess the effect of changes in protein net charge on adsorption to charged silicon oxide surfaces. A range of adsorption and rinse steps were implemented to determine the robustness of the adsorbed protein layer(s). IgG1 that was desorbed from the sample cell was analysed using various methods in order to quantify aggregated material. The results suggested that exposure to the silica surface caused increased quantities of aggregate species to form, provided IgG films were able to desorb under low Reynolds-number wash steps. Independent of the wash steps, the reflectivity data indicated that IgG1 adsorbed to the surface in a largely flat-on orientation with a high level of surface contact.

Another important class of antibodies in biotherapeutics is the IgG4 class [4]. The relatively short length of the hinge in IgG4 has been shown to restrict movement of the Fab regions, giving a predominantly asymmetric conformation [4]. IgG binds protein A via its Fc region. Staphylococcal protein A contains five IgG-binding domains, but steric restrictions mean that a single protein A molecule will only bind up to two IgG molecules at once. The domains of protein A are arranged in series, giving a flexible chain-like structure [14]. Due to the relatively limited flexibility of IgG4 about its hinge we expect that IgG will adopt a more surface-distal or upright orientation when bound to protein A, rather than the likely flat-on orientation during adsorption directly to silica.

In this work we will build on the work of Perevozchikova et al. [11], and others [8], [12], [15], by using neutron reflectivity to determine the structure of IgG4 adsorbed to silica under protein A chromatography buffer conditions. Data for IgG4 adsorbed directly to silica will then be used as a control with which we can compare the more complex model system that is IgG4 adsorbed to immobilised protein A.

## 2 Neutron reflection theory

A useful property of neutrons is that their interactions with each of the hydrogen isotopes protium (^1^H) and deuterium (^2^H) are distinct. The property *neutron scattering length* describes a material’s ability to scatter neutrons; deuterium has a large scattering length while that of protium (99.985% natural abundance hydrogen) is negative [6]. When measuring bulk materials or large structures like proteins, scattering lengths of all the atoms in a given molecule are summed and divided by the volume of the molecule to give the quantity *neutron scattering length density* (nSLD) which has units Å^−1^. It follows that the nSLD of H_2_O is negative, while that of D_2_O is positive and relatively large. Neutrons are scattered at interfaces between materials of differing nSLDs; the magnitude of this scattering is determined by the difference in nSLD across the interface. This difference in nSLD between two bulk phases or materials is often termed *contrast* [5].

**Fig. 1:**
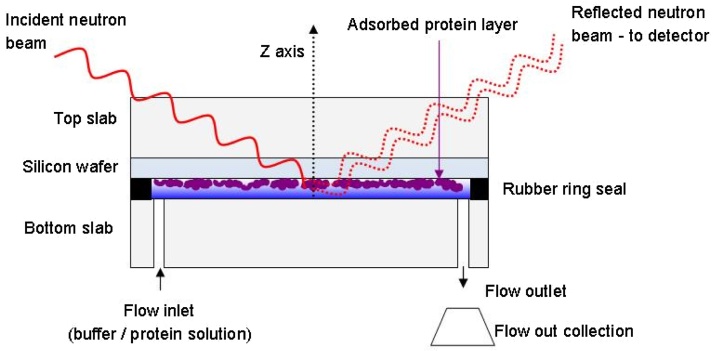
Schematic representation of the flow cell used for reflectivity experiments. The flow-through cavity has a volume of approximately 1.5 mL and a height of 0.1 mm. During experiments all space inside the cell is filled with aqueous solution at all times. For a bare silicon surface the incident angle of the collimated neutron beam is equal to the reflected angle. Neutrons reflect at interfaces present inside the reflectivity cell, such as the SiO_2_-protein interface and the protein-water interface. The reflectivity profile generated by adsorbed material depends on five major factors: its chemical composition, the number of layers present across the z axis, their respective depths and volume fractions, and the resolution or smoothness of transition between layers (roughness).

Fig. 1 schematically depicts the *solid-liquid flow cell* (SLFC) used to hold the sample in reflectivity experiments. The silicon wafer, onto which sample molecules adsorb, is a flat, highly polished surface. A highly collimated beam of neutrons is passed into the sample cell from above at a very low grazing angle, resulting in reflection of the beam at the solid-liquid interface. This reflectivity is determined by the incident angle of the neutron beam, the neutron wavelength, the nSLD difference between the bulk phases, and the size and structure of any materials present at the interface.

The neutron wave can be described in terms of a wavevector $\vec{k}$, having a magnitude and a direction. The wavevector $\vec{k}$ points along the neutron’s trajectory and has a magnitude

(1) $$\vec{k}=2\pi /\lambda$$

where λ is the neutron wavelength.

When the incident wave is reflected from the surface momentum transfer occurs. The wavevector transfer in the z direction, perpendicular to the sample surface, is denoted Qz. For specular reflectivity $\vec{k_{r}}=\vec{k_{in}}$. Therefore

(2) $$Q_{z}=k_{in\;z}-k_{r\;z}=\frac{2\pi }{\lambda }\left(\sin\;\theta_{in}+\sin\;\theta_{r}\right)=\frac{4\pi \;\sin{\;\theta }_{\mathrm{in}}}{\lambda }$$

where *k_inz_* and *k_rz_* are the *z* components of the incident and reflected neutron wavevectors, respectively; *θ_in_* and *θ_r_* are the angles of incidence and reflection (which are equal for specular reflection), respectively [5]. *Q_z_* has units of Å^−1^ and is often abbreviated to simply *Q*.

In Fig. 1 the red dotted lines representing the reflected beam show how neutrons are reflected at the upper and lower interfaces of a thin film. When waves are reflected at parallel interfaces interference patterns can appear. Constructive interference occurs when reflected waves remain in phase. Conversely, if reflected waves become out of phase the interference is destructive [5]. Reflectivity data is initially presented as reflected neutron intensity as a function of Q_z_. The presence of a thin film at the solid-liquid interface affects the profile of reflectivity against Q_z_ such that interference fringes (dips or peaks in reflectivity intensity) appear [6]. The distance in Q_z_ between fringes is related to the thickness of the film by

(3) *Q_z2_* − *Q_z1_* = *2π/d*

where *Q_z2_ *− *Q_z1_* is the distance in Q between the fringes (Å^−1^) and *d* is the thickness of the interfacial film (Å) [5], [6].

Though films with depths of less that 35 Å do not produce highly pronounced fringes in reflectivity profiles, the SLD of the film relative to other phases significantly impacts the shape of the reflectivity profile [6]. Implementing a contrast variation approach to experimental design allows complex information to be obtained even for very thin films and sparse layers of material. One simple approach is to use D_2_O as a mobile phase when measuring hydrogenous structures like protein, as implemented here.

## 3 Materials and methods

### 3.1 Materials

The IgG4 was kindly donated by UCB Celltech (UCB Celltech, Slough, UK). It is a purified IgG4 kappa antibody; the hinge region is not mutated. The IgG was formulated at 17.8 mg/mL in 270 mM glycine, 1% maltose, pH 5.0. This IgG4 kappa has an isoelectric point between 6.85 and 8.15 and a molecular weight of 165 kDa.

Silicon wafers for reflectivity at the NIST Center for Neutron Research (NCNR) were from El-Cat (New Jersey, USA); other NCNR reflectivity cell components were custom made for laminar flow reflectometry.

Silicon wafers and solid-liquid flow cells (SLFCs) for reflectivity at the ISIS Neutron and Muon Source were purpose built. 3-aminopropyltriethoxysilane (APTS) and sulfosuccinimidyl 6-(3′-(2-pyridyldithio)propionamido) hexanoate (sulfo-LC-SPDP) were from Thermo Scientific Pierce (Thermo Fisher Scientific, Leicestershire, UK).

Recombinant staphylococcal protein A was from Repligen (Waltham, MA, USA). Deuterium oxide (99.9 atom % D) and bovine serum albumin (fraction V) were from Sigma-Aldrich (Dorset, UK); PEG_6000_ was from VWR Chemicals (Leicestershire, UK). Extra dry acetonitrile was from Fisher (Leicestershire, UK).

### 3.2 Equipment

Attenuated total reflectance Fourier transform infrared spectroscopy (ATR-FTIR) was conducted using a Thermo Nicolet Nexus instrument (Madison, WI, USA) that was fitted with a monolayer/grazing angle accessory (Specac 19650 series, Kent, UK), a mercury cadmium telluride detector, and an air dryer to purge water vapour and carbon dioxide from the instrument [16].

Reflectivity measurements at the NCNR (Gaithersburg, MA, USA) were performed on the NG7 horizontal neutron reflectometer. Reflectivity measurements at the ISIS Neutron and Muon Source (Rutherford Appleton Laboratory, Harwell Science and Innovation Campus, Oxfordshire, UK) were performed on the INTER time-of-flight reflectometer. Buffer flow through the SLFCs was achieved using an L7100 HPLC pump from Merck-Hitachi (Kent, UK).

### 3.3 Methods

#### 3.3.1 Reflectivity experiments at the NIST Center for Neutron Research

##### 3.3.1.1 Sample preparation

IgG4 was prepared by dialysis into in H_2_O-based 0.03 M sodium citrate, pH 4.03 and pH 6.21, and D_2_O-based 0.03 M sodium citrate, pH 4.10 and pH 6.24. Each dialysis was carried out in three stages, resulting in a final dilution factor of at least 3000 for original buffer constituents. The IgG concentration in the samples was determined by measuring the absorbance at 280 nm using a NanoDrop 1000 spectrophotometer (Thermo Scientific, Wilmington, DE, U.S.A.), and using Beer’s law and an extinction coefficient $\varepsilon_{1cm}^{0.1\%}=1.61$ (experimentally determined by UCB Celltech, Slough). All samples were adjusted to an IgG4 concentration of 9.5 mg/mL. Before injection into the reflectivity cell, samples were filtered through a 0.2 μm membrane and diluted in their respective buffers to a final IgG concentration of 5.6 mg/mL.

##### 3.3.1.2 Reflectivity cell

The reflectivity cell was prepared using the method described by Perevozchikova et al. [11]. In brief: circular silicon wafers approximately 76 mm in diameter and 5000 μm thick were cleaned using a Piranha etch solution and rinsed with ultrapure water before assembly into the SLFCs. The assembled reflectivity cell contained a chamber, created by a Viton spacer, with a volume of approximately 1.5 mL and a height of 100 μm [11].

##### 3.3.1.3 Reflectivity experiments

The reflectivity cell was placed on the sample stage and connected to a syringe pump with valved tubing such that sample changes could be made without introducing air into the cell. Two separate reflectivity cells were used for the experiments; at the beginning of experiments each cell was aligned and slit scans were taken.

Measurements were taken at Q_z_ values ranging from 0.008 Å^−1^ to 0.250 Å^−1^. For each sample configuration, neutron data collection took place over a minimum of 6 h; this provided sufficient counting statistics to resolve signal over background counts [11]. For each sample configuration background scans were programmed to run periodically between sample scans; background scans ran over a short time period (20 min each) for a selection of Q values across the full Q range. All scans were run in duplicate and in some cases triplicate. The reflectivity instrument counts the number of neutrons that hit the detector for a given Q value. The counts are normalised based on an intensity of 1 for total internal reflection of the neutron beam. For each scan, error bars equal the square root of the number of counts before normalisation.

The first SLFC was washed with 7.5 mL buffer and neutron measurements were made for the bare silicon wafer for two solution phase contrast conditions, D_2_O and H_2_O. In the first SLFC, after equilibration with D_2_O-based buffer, 3 mL IgG4 in 0.03 M sodium citrate, pH 4.1 was flowed into the reflectivity cell at 6 mL/h. The IgG solution remained in the cell during reflectivity measurements. Reflectivity data was subsequently acquired after rinsing with D_2_O-based buffer, after IgG re-adsorption in the H_2_O solution phase contrast, and after rinsing with H_2_O-based buffer.

In the second SLFC, reflectivity data was obtained in D_2_O and H_2_O solution phase contrasts for the following sample configurations: IgG adsorbed in 0.03 M sodium citrate, pH 6.2; IgG solution washed out with the same buffer; surface washed with 0.03 M sodium citrate, pH 3.7.

##### 3.3.1.4 Data analysis

Initial data processing was done using Reflred software, and NCNR in-house software described elsewhere [17]. Low Q and high Q scans were stitched together and averages taken for replicate scans. Slit and background scans were averaged and subtracted from the stitched sample scans to give the final reflectivity curves.

Model fitting was done using RasCal software [18] which fits Abeles layer models to the data using an optical matrix formalism [19]. Fitting error analysis was performed using the *Bootstrap error analysis* function in RasCal [18]. Models were selected based on a combination of lowest chi-squared values and most robust key fitted parameters (results of the bootstrap error analysis). For a description of how models were constructed, see Clifton et al. [6]. Where data for a given sample configuration was collected at more than one solution-phase contrast, multiple contrasts were fitted simultaneously to a single layer depth profile. Though there is some evidence that D_2_O-based buffers can influence protein-protein interactions and phase behaviour [20], it was assumed that structures were effectively the same across the different contrasts.

#### 3.3.2 Experiments at the ISIS Neutron and Muon Source

##### 3.3.2.1 Sample preparation: protein A

Recombinant staphylococcal protein A (rSPA) was dialysed into D_2_O-based *PBS-EDTA* buffer, pH 7.4, in two stages with a total dilution factor of 100,000 for original buffer constituents. Recombinant-SPA concentration was determined using Beer’s law and a percent solution extinction coefficient *ε*^1%^ = 1.65 (275 nm). LC-SPDP was attached to rSPA by following the protocol in the product manual from Thermo Fisher Scientific, this is based largely on methods by Carlsson et al. [21]. The linker-modified rSPA was separated from reaction by-products using PD-10 Desalting Columns (GE Healthcare, Buckinghamshire, UK). The molar ratio of protein to linker was determined using a method by Carlsson et al. [21].

##### 3.3.2.2 Sample preparation: IgG

IgG4-κ was dialysed into D_2_O-based 0.03 M sodium phosphate, pH 6.7, in two stages with a total dilution factor of 100,000 for original buffer constituents. Dialysed material was prepared at 18 mg/mL IgG4 and stored at 4 °C for up to 5 days. The dialysed IgG was diluted to 1 mg/mL in the same buffer for FTIR and reflectivity experiments.

##### 3.3.2.3 ATR-FTIR experiments

The silicon crystal was cleaned by ozone cleaning, creating a highly hydrophilic surface. The crystal was amino-silylated by immersion in a 2% solution of APTS in extra dry acetonitrile for 30 s; it was then rinsed with extra dry acetonitrile and allowed to dry. The amino-silylated crystal was assembled in a solid-liquid flow cell custom made to fit the FTIR instrument. All subsequent surface modifications were carried out in flow-through mode. Flow was controlled by a peristaltic pump at a flow rate of approximately 1 mL/min. For reagent additions at least 5 flow-cell volumes and for rinse steps at least 8 cell volumes were used. All FTIR experiments were carried out using D_2_O-based buffers.

Modified rSPA was cross-linked to the amino-silylated surface using methods described by Carlsson et al. [21]. The cross-linking reaction was allowed to proceed overnight. After rSPA cross-linking the surface was incubated with 1% BSA solution. The buffer was changed from PBS-EDTA to 0.03 M sodium phosphate, and a new background was taken before addition of 1 mg/mL IgG4. The IgG solution was rinsed out after 10 min. IgG was eluted with 0.1 M Glycine-HCl, pH 4.1, and then pH 3.7.

FTIR data collection and analysis was accomplished using Omnic software (Thermo Scientific), using the same methods as Clifton et al. [22].

##### 3.3.2.4 Reflectivity experiments

Rectangular silicon wafers (50 × 80 × 20 mm; 20 mm = thickness) were cleaned using the method described in Section 3.3.1.2 and amino-silylated using the method described in Section 3.3.2.3. Three separate SLFCs were used, *cells A*, *B*, and *C*. In all cases, the silicon wafers were treated with APTS, producing an amino-silylated surface, before assembly into SLFCs; subsequent surface modifications were carried out in flow-through mode. Reflectivity measurements were taken at Q_z_ values ranging from 0.01Å-1 to 0.20Å-1. Glancing angles of 0.7° and 2.3° were used to acquire data in the Q ranges 0.01–0.1Å-1 and 0.033–0.20Å-1, respectively.

Each sample cell was analysed before any surface modifications were made (apart from amino-silylation). Reflectivity data was collected during and after various stages of surface modification such as cross-linker attachment, rSPA cross-linking, surface blocking, IgG adsorption, IgG elution and surface regeneration. Cross-linking was done under conditions described in Section 3.3.2.3. The buffer was changed to 0.03 M sodium phosphate, pH 6.7 (IgG adsorption buffer) before neutron measurements were made. In two of the SLFCs PEG_6000_ was used as a blocking molecule instead of BSA. For protein-containing sample configurations, data was collected for three solution phase contrasts: D_2_O, H_2_O and *silicon matched water* (SMW). SMW contains 38% D_2_O and 62% H_2_O.

Initial data processing was done using MantidPlot [23]. The data was processed similarly to that collected at the NCNR (Section 3.3.1.4). Background subtraction was not implemented for the raw data and was instead integrated into the model fitting process using RasCal [18].

## 4 Results and discussion

### 4.1 The conformation of IgG4 adsorbed to silica at pH 4.1

In the first part of this work neutron reflection was used to probe the structure and orientation of IgG4 adsorbed to the model surface silica.

IgG4 adsorption and rinse steps were carried out and reflectivity measurements were taken in D_2_O and H_2_O-based buffers. In a typical experimental cycle, IgG was initially adsorbed inside the reflectivity cell in D_2_O-based buffer. Neutron measurements were made during adsorption and after rinsing with D_2_O-based buffers of interest. To obtain corresponding data in the H_2_O contrast, IgG in H_2_O-based buffer was introduced into the same reflectivity cell (without cleaning), and subsequent measurements and buffer rinses were carried out. We note that *elution* of adsorbed IgG was not carried out, only rinsing, before re-application of IgG in the H_2_O contrast. The factor that differentiates the *adsorption* configuration from the *rinse* configuration across both contrasts is the presence of IgG solution in the SLFC during neutron measurements.

**Fig. 2:**
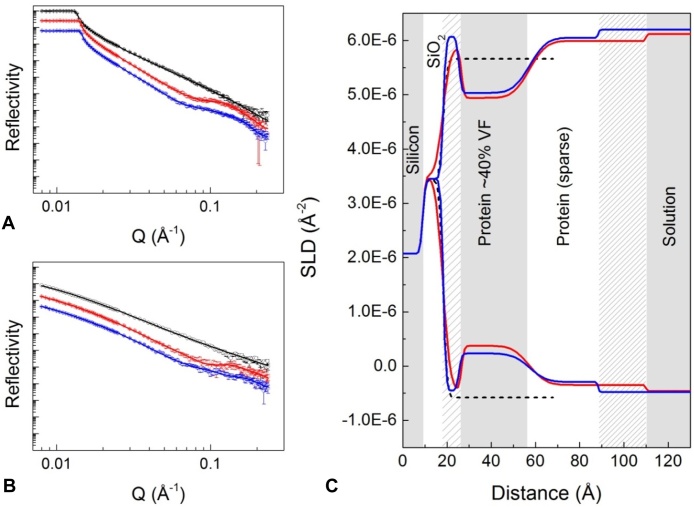
Left-hand plots (A) and (B): reflectivity (relative intensity) against momentum transfer vector, Q (Å^−1^), for sequential IgG adsorption and wash steps carried out in D_2_O based (A) and H_2_O based (B) sodium citrate buffer. In all cases H_2_O buffer was at pH 4.00 and D_2_O buffer was at an apparent pH of 4.10, which approximates to pD 4.5. Data sets are offset down the y-axis for clarity; in D_2_O the critical edge always occurs when reflectivity is equal to unity. Different colours represent different experimental conditions/stages. From top to bottom: black is the bare silicon wafer; red is IgG adsorption at 5.6 mg/mL; blue is adsorbed IgG after a 6.0 mL rinse. Solid lines show the fitted models. Plot (C) shows the corresponding profiles of scattering length density, SLD (Å^−2^), against distance from the silicon surface, each generated by simultaneous fitting of D_2_O and H_2_O reflectivity data sets. Curves that terminate at the upper and lower ends of the SLD scale represent D_2_O and H_2_O data, respectively. (For interpretation of the references to colour in this figure legend, the reader is referred to the web version of this article.)

Illustrative examples of reflectivity profiles as a function of Q (wavevector transfer in the z direction, with units Å^−1^) are shown in Fig. 2. Panel (a) shows IgG adsorption and rinsing with D_2_O-based buffer at the silicon-liquid interface, and panel (b) shows the corresponding measurements made in H_2_O-based buffer. In both cases, black points are for the bare silicon wafer immersed in buffer. The intensity of reflection at the silicon-H_2_O interface is around an order of magnitude lower than that in the D_2_O liquid phase. This is expected because there is a smaller difference in SLD between (hydrogenated) protein and H_2_O than between protein and D_2_O. Nonetheless, the H_2_O data has an important role in the model fitting process because simultaneous fitting of D_2_O and H_2_O data sets gives a higher level of confidence in the fit. Looking at the shapes of the reflectivity profiles for IgG adsorption and rinse stages in Fig. 2a and b, significant deviation from the silicon-water only interface can be seen.

Fig. 2c shows the SLD profiles as a function of z-direction distance, corresponding to the fitted curves in Fig. 2a and b. Models consist of a series of slabs/layers building in the z-direction; the SLD of each layer depends on the material present (e.g. protein or silicon) and it’s volume fraction in the solvent. Models also contain a “roughness” parameter, defined by whether transitions between layer SLDs are sharp or gradual. For all data sets, a layer of silicon oxide was included in the model; this layer homogenously coats the silicon substrate, so the SLD value was constrained closely around the predicted value of 3.43 × 10^−6^ Å^−2^ for both H_2_O and D_2_O solution phase contrasts. The thickness of the layer was allowed to vary and typically fell close to 10 Å. Looking at the wafer-only curve in Fig. 2c, we can see that the SLD of the D_2_O bulk solvent is lower than that of the other curves; this corresponds to a shift in critical edge (Fig. 2a), and was attributed to the presence of a small quantity of H_2_O in the buffer.

When IgG was adsorbed, the lowest chi-squared values were obtained when the model included three distinct layers in addition to SiO_2_. Note that these can be considered hypothetical layers and could represent sub-structures of a monolayer. Initially, a two-layer model was fitted to the data, that is, two layers of protein after SiO_2_. This gave reasonable chi-squared values but suggested an unrealistically dense layer of protein adjacent to silica (fit not shown). Using a similar approach to Couston et al. [24], an additional layer was added to the model; this resulted in a lower chi-squared value and improved physical feasibility. This layer can be seen in Fig. 2c after the SiO_2_ layer and will henceforth be denoted *wafer-proximal layer*. The subsequent layers in the z direction are denoted *inner protein* and *outer protein*. The fitting outputs are summarised in Table 1. We suggest that the wafer-proximal layer represents the part of the protein hydration shell that is adjacent to the solid interface.

SLD values were used to estimate adsorbed material volume fraction using the following equation:

(4) $$\phi_{l}=\left(\rho_{s}-\rho_{l}\right)/\left(\rho_{s}-\rho_{a}\right)$$

where *ϕ_l_* is the volume fraction of adsorbed material, *ρ_s_* is the SLD of the solvent, *ρ_l_* is the fitted SLD of the layer and *ρ_a_* is the calculated SLD of the (pure) adsorbed material [22]. The inputs and outputs of volume fraction calculation are shown in Table 1. These volume fraction values assume 90% of labile hydrogens were exchanged to deuterium. The value for volume fraction obtained in this way does not account for tertiary structure of the adsorbed material. This is significant for proteins because the tertiary structure may incorporate solvent-filled cavities and highly protected hydrogen bonds [25].

During IgG adsorption the wafer proximal, inner protein, and outer protein layers had thicknesses of 8 Å, 31 Å and 53 Å, respectively (Fig. 2c). The inner protein layer had the highest volume fraction, estimated at 0.40, while the wafer-proximal and outer protein layers had lower volume fractions of approximately 0.05 each (Table 1). After rinsing, the inner protein layer remained much the same as before, while the outer layer displayed a significant decrease in depth from 53 Å to 31 Å (Fig. 2).

To interpret the data in terms of IgG orientation, one can consider the individual layer thicknesses and also the total thickness of adsorbed material and compare these to related values in the literature; we keep in mind that the adsorbed material may be in a dynamic state. Abe et al. [26] and Rayner et al. [4] used x-ray and neutron scattering methods to examine the structure of IgG4 in bulk solution [4], [26]. IgG4 was found to have a maximum length of 170 Å. R_g_, R_xs-1_ and R_xs-2_ values were also determined. For an ellipsoid, the mean radius of gyration of the two shorter axes is denoted R_xs_, while R_g_ gives the average about all three axes. For immunoglobulins, R_xs_ is split into two components, R_xs-1_ and R_xs-2_. The average cross-sectional radius is described by R_xs-2_, and R_xs-1_ is a measure of Fab and Fc separation [4], [27]. Rayner et al. [4] determined R_g_, R_xs-1_ and R_xs-2_ values of 48 Å, 25 Å and 12 Å, respectively, from neutron data [4]. These values are comparable to the protein layer thicknesses seen in Fig. 2c. For example, doubling R_xs-2_ gives a 24 Å average cross-sectional diameter, which is a little smaller than the 31 Å inner protein layer reported here (Fig. 2c). We hypothesise that this inner protein layer represents a majority population of IgG molecules adsorbed in a “flat on” orientation, i.e. IgG with its small cross-section perpendicular to the surface. The volume fraction of the outer protein layer is approximately 10 times smaller than that of the inner layer. With a thickness slightly greater than double R_xs-1_, this outer layer could represent a sparse self-associating IgG layer in a flat to side-on orientation. After rinsing, the depth of the outer layer decreases by 22 Å while the volume fractions are barely altered (Table 1). This suggests that the outer layer is more weakly adsorbed and undergoes re-organisation on rinsing. Another possibility is that the outer layer seen during the adsorption stage constitutes an occasional Fab arm extending outwards. Similar interpretations can be made when comparing the data to the dimensions of the IgG4 crystal structure obtained by Scapin et al. [28].

When adsorbed proteins demonstrate a flat-on orientation, the calculated volume fraction values can be used to estimate the *surface coverage* that is, the mass of adsorbed material per unit area, which may in turn be used to estimate the average area occupied by each adsorbed protein molecule. This lends insight into surface packing density [12]. The following equation is used to estimate surface coverage, Γ

(5) $$\Gamma =\phi_{l}.\tau .\rho {\text{'}}_{p}$$

where *ϕ_l_* is the volume fraction (see Eq. (4)), *τ* is the layer thickness (cm) and *ρ'_p_* is the protein mass density (g cm^−3^) [8]. The protein mass density of the IgG was assumed 1.42 g cm^−3^ [8]. If the inner layer of IgG adsorbed at pH 4.1 is assumed to represent a monolayer of IgG in a flat-on orientation, the surface coverage approximates to 1.7 mg m^−2^. From this mass coverage value, we can calculate the number of molecules per square metre, and then determine approximately how much excess space surrounds each molecule (assuming homogenous coverage). For IgG4 with a molecular weight 165 kDa, 1.7 mg m^−2^ equates to an area of 16120 Å^2^ per IgG molecule. The minimum area required for an IgG4 to adsorb flat-on is approximately 13,600 Å^2^ [4]. This suggests a closely packed layer of adsorbed proteins with each IgG molecule given around 1.2 times its minimum required area.

### 4.2 The influence of pH on IgG4 adsorption to silica

**Fig. 3:**
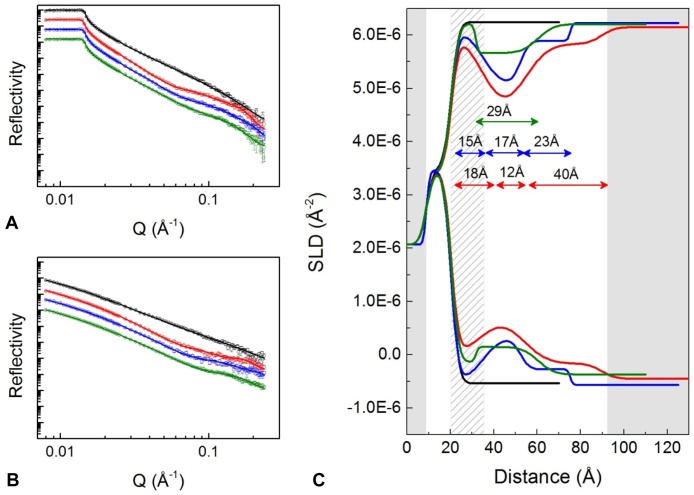
Reflectivity data for sequential IgG adsorption and wash steps carried out in D_2_O based (A) and H_2_O based (B) sodium citrate buffer. From top to bottom: black is the bare silicon wafer; red is 5.6 mg/mL IgG adsorption at pH 6.2; blue is adsorbed IgG after a 6.0 mL rinse with pH 6.2 buffer; green is after a subsequent 6.0 mL rinse with pH 3.7 buffer. Solid lines show the fitted models. Plot C shows the corresponding profiles of scattering length density, SLD (Å^−2^), against distance from the silicon surface. Layer thicknesses in each SLD-distance profile are shown by double-ended arrows. (For interpretation of the references to colour in this figure legend, the reader is referred to the web version of this article.)

Fig. 3 shows neutron data for IgG adsorption to silica at pH 6.2, adsorbed IgG structure after rinsing at the same pH, and after rinsing at pH 3.7. A three-layer model, similar to that for pH 4.1, was fitted to the data. For clarity’s sake we will assume the same naming convention as above – wafer-proximal, inner protein and outer protein layers. During pH 6.2 adsorption the three layers had a total thickness of 70 Å, the maximum volume fraction of the inner protein layer was 0.45, and those of the wafer-proximal and outer protein layers were 0.16. Notable differences from pH 4.1 data include higher volume fractions, higher roughness between layers and a thicker wafer-proximal layer. Here, we hypothesise that the wafer-proximal layer also constitutes a region of the IgG, due to its greater depth, volume fraction and roughness, compared to the wafer-proximal layer at pH 4.1. The data suggests a tilted to side-on orientation of IgG, where one Fab arm may partially contact the surface while the other extends into the solution. Xu et al. [8], [30] determined a very similar three-layer structure for an IgG1 adsorbed to silica at pH7.0 [8]. In this work, the higher volume fractions at pH 6.2 compared those at pH 4.1 suggest closer packing together of IgG molecules, and increased roughness indicates a less defined orientation, and so possibly weaker adsorption to the interface. These are expected findings for an increase in pH towards the protein’s isoelectric point, in this case ∼pH 7.5 [8], [9], [29]. Indeed, after rinsing at the same pH, volume fractions for all layers were reduced, indicating loss of adsorbed material. The total thickness decreased from 70 Å to 55 Å. When the buffer pH was reduced from 6.2 to 3.7, the shape of the profile reverted to one similar to that seen at pH 4.1, but lacking the outermost layer. Here, the SLD of the wafer-proximal layer matches that of the bulk solvent, indicating that the wafer-proximal layer constitutes a hydration layer. The low roughness between the two layers, and the lack of an outer protein layer, suggests that the IgG became adsorbed in a completely flat on orientation when the pH was lowered. The apparent high level of surface contact could be due to electrostatic attraction between positively charged IgG molecules and the negatively charged silica surface. The data highlights the importance of protein-protein charge repulsion in determining the packing density of interfacially adsorbed proteins, that is, packing density declines as protein charge increases, even though electrostatic attraction between protein and surface may have increased, supporting the findings of various other studies [8], [9], [11], [13], [30]. There is also the matter of charge screening, which increases with buffer molarity, but in this case all buffers had the same molarity. Finally, we note that charges on different regions of an IgG, e.g. Fc or Fab, can differ significantly from the net charge [31], [32], and could, therefore, influence molecular orientation at the interface with pH-dependence.

### 4.3 Protein A chromatography surface mimic

To extend the study towards understanding the structure of IgG adsorbed on affinity chromatography resins, protein A was covalently attached to a silicon wafer. The protein A immobilisation process was initially monitored using attenuated total reflectance Fourier transform infrared spectroscopy (ATR-FTIR). Subsequently, neutron reflectivity was used to examine protein structure on the surface at various stages of protein A immobilisation and IgG adsorption.

Recombinant staphylococcal protein A (rSPA) contains five approximately 7 kDa IgG-binding domains and a truncated *X_t_* domain. The six domains are arranged in sequence resulting in a long, flexible chain-like protein [14], [33]. The heterobifunctional cross-linker used to attach rSPA to the silica surface reacts with proteins via primary amine groups, i.e. lysine residues [34], [35]. We note that some lysine residues are present on the IgG-binding domains (Supplementary Fig. S1): one lysine on helix II is indicated in, but not essential to, IgG-binding [36], [37]. An abundance of lysine residues are present on the *X_t_* domain (Supplementary Fig. S1b). Using a spectrophotometric method [21], the molar ratio of cross-linker to rSPA was found to be low at 2:1. As rSPA has five IgG-binding sites we made the assumption that IgG binding was not significantly affected by the presence of excess cross-linkers.

### 4.4 Monitoring surface modifications with ATR-FTIR

**Fig. 4:**
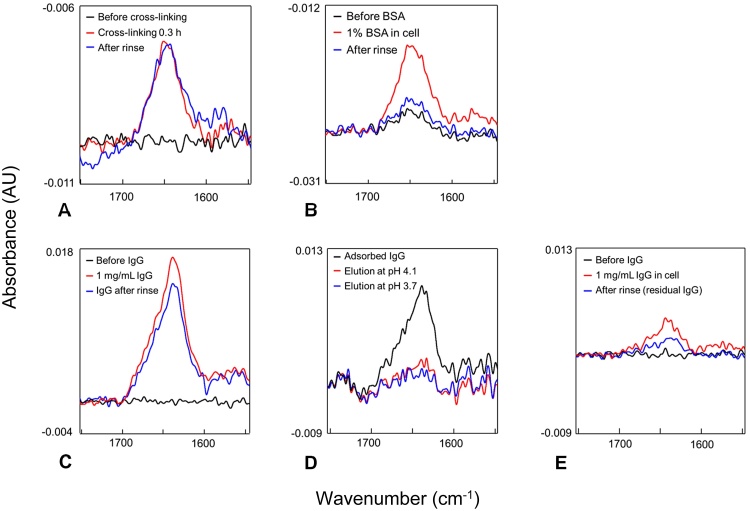
FTIR spectra for sequential stages of silica surface modification, IgG adsorption and elution. Within each plot, the event sequence is analogous to the following colour sequence: black (initial) – red – blue (end). The instrument was blanked and SPDP-modified rSPA was introduced into the cell; reaction with cross-linkers on the silica surface resulted in cross-linked rSPA (A); BSA was then used to block un-reacted or “sticky” sites (B). The cell was equilibrated in adsorption buffer, the instrument was blanked and IgG was introduced (C). Adsorbed IgG was eluted first at pH 4.1 and then at pH 3.7 (D). Cross-linked rSPA was cleaved by DTT reduction and reactive cross-linkers were blocked with denatured BSA; IgG was then introduced to check for non-specific binding (E). (For interpretation of the references to colour in this figure legend, the reader is referred to the web version of this article.)

ATR-FTIR spectra for various stages of surface modification and IgG4 binding and elution are presented in Fig. 4. The sample environment was designed to be close to that of the reflectivity experiments, including use of D_2_O-based buffers, making it a useful orthogonal measurement technique.

Spectra in Fig. 4 focus on changes in the wavenumber range 1600–1700 cm^−1^ because the major protein-related band, amide I, lies in this region [38]. When SPDP-modified rSPA was attached to the surface, the size of the amide I peak after 18 min of reaction time was the same as that after excess SPDP-rSPA was rinsed from the sample cell (after overnight incubation) (Fig. 4a). This indicates that the reaction took place rapidly and long incubation times were not necessary. In Fig. 4b a slight increase in the height of the amide I peak can be seen after BSA blocking. During incubation with 1% BSA a large peak was seen because of the high concentration of BSA (10 mg/mL); however, after rinsing it was found that only a small quantity of BSA remained at the interface.

Incubation of IgG4 with the rSPA-modified surface resulted in a large amide I peak that persisted after removal of IgG solution from the sample cell by rinsing with several cell-volumes of buffer (Fig. 4c). The adsorbed IgG was then eluted by rinsing first at pH 4.1 and then at pH 3.7; most of the IgG eluted at pH 4.1 (Fig. 4d). This elution pH is slightly higher than would typically be expected for elution of IgG from protein A [39], [40], [41], [42]. However, recent work by Gagnon and Nian has shown that IgG may interact with protein A by a dual-site binding mechanism in typical chromatography *column* environments [3], with “single site bound” IgG populations eluting at a higher pH. Thus, it is possible that the packing density of rSPA crosslinked to the surface was such that each IgG4 molecule bound a single protein A ligand, resulting in near-total elution at pH 4.1. Another consideration is the potential impact of D_2_O-based buffers on protonating behaviour [43], with possible effects on both binding affinity and buffer properties [20].

Non-specific attachment of rSPA was investigated by incubating rSPA and SPDP-rSPA with an APTS-coated silica surface; minimal non-specific adsorption of rSPA was observed (data not shown). To test for non-specific binding of IgG, BSA was cross-linked to the surface instead of rSPA before IgG incubation in the sample cell. A very small amide I peak was observed for non-specifically adsorbed IgG, as can be seen in Fig. 4e. Rinsing with pH 3.7 buffer eliminated this amide I peak (data not shown). Therefore, the peak was attributed to IgG interacting with non-specifically adsorbed rSPA, which was present on the surface from previous incubations with rSPA.

FTIR spectra for cross-linked rSPA and adsorbed IgG4 were integrated within the amide I and amide II regions and the peak areas were compared. The IgG peak area was 2.5 times larger than the rSPA area (data not shown). This is suggestive of a 1:1 ratio of IgG: rSPA, as the IgG is approximately 3 times the molecular weight of rSPA. Although the data include significant noise, analysis of the sub-peaks in amide bands suggests that each protein’s characteristic secondary structure content was reasonably maintained on immobilisation/adsorption, that is, mostly alpha helix and random coil for protein A, and mostly beta sheet for IgG (analysis not shown). The peak designations were valid for proteins in D_2_O-based solutions [38], [44], [45].

### 4.5 Neutron reflectivity measurements of protein A-IgG complex at interface

A summary of the neutron data collected and chi-squared values for models fitted using data from one two or three solution-phase contrasts, across three sample cells, is given in Table 2. In all three cells protein A was cross-linked to the surface, a blocking agent was applied and IgG was adsorbed. In cell A BSA was used as a blocking agent, and in cells B and C PEG_6000_ was used.

#### 4.5.1 Protein A/BSA surface characterisation

**Fig. 5:**
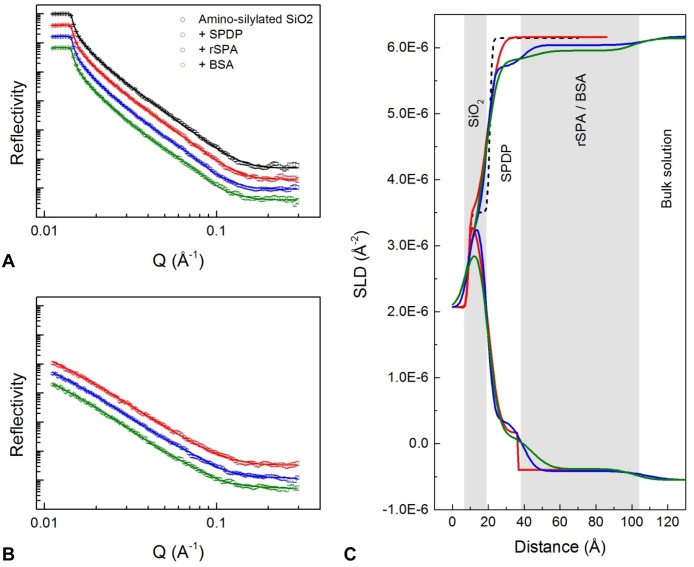
Reflectivity data for attachment of rSPA to an amino-silylated silica surface, and addition of the blocking agent BSA. Left-hand plots A and B: reflectivity (intensity) against Q (Å^−1^) in D_2_O-based (A) and H_2_O-based (B) buffer at various stages of surface modification. From top to bottom: black is the amino-silylated wafer; red is with the cross-linker attached, blue is after rSPA cross-linking and green is after blocking with BSA. Solid lines show the fitted models. Plot C shows the corresponding profiles of scattering length density, SLD (Å^−2^), against distance from the silicon surface. (For interpretation of the references to colour in this figure legend, the reader is referred to the web version of this article.)

The reflectivity profiles for the modified surface in Fig. 5a and b (cell A) do not show large deviation from the APTS-coated surface. However, as material was added to the surface, reflectivity in the Q region approximating 0.02–0.1 Å^−1^ shifted to slightly lower values. The largest shift occurred on rSPA crosslinking. The SLD profiles from the model fits are shown in Fig. 5c. When only the cross-linker was on the surface, a 15 Å thick SPDP layer gave the best fit. The model for cross-linked rSPA indicated a 20 Å thick layer proximal to SiO_2_, and 69 Å thick layer beyond this. The 69 Å layer was attributed to rSPA. Using Eq. (4), a surface volume fraction of 5% was estimated. This initially seems low coverage. However, coverage can appear low if long thin proteins are orientated with their length extended in the surface normal direction [11].

Capp et al. [14] found the R_g_ of a single protein A domain to be 13 Å and the persistence length to be 36 Å for five protein A domains in sequence. The persistence length is related to R_g_ and provides a characteristic length scale of chain flexibility in polymer models [14]. The modelled depth of the rSPA layer is slightly less than double the persistence length for protein A determined by Capp et al. [14]. In this work staphylococcal protein A was used (Supplementary Fig. S1), the *X_t_* domain of which is expected to contribute to the total protein length. Protein A may be less flexible in the immobilised state, compared to in free solution, and the presence of excess SPDP ligands could contribute to a reduction in flexibility. Thus, 69 Å seems a reasonable value for the protein A layer thickness. Using Eq. (5) the surface coverage of protein A was estimated to be 0.47 mg m^−2^; this equates to approximately 17,100 Å^2^ space per rSPA molecule for an evenly dispersed layer. Assuming upright rSPA molecules with cross-sectional areas of 26 × 26 Å, each rSPA molecule is estimated to occupy an area large enough for ∼25 rSPA molecules. If the cross-sectional area is assumed larger, due to a tilted or flexing orientation, the excess surface space may reduce to around 10 molecules. If we take the square root of 17,100, we get a length of 131 Å. This is a similar value to the expected total length of protein A.

We can make a rudimentary comparison between this protein A coverage (mg m^−2^) and that of a commercial chromatography resin, such as Millipore’s Prosep Ultra Plus, by making calculations based on the resin dynamic binding capacity (DBC), 48 mg/mL [46], and average bead size, 60 μm. If we assume a 1:1 ratio of IgG to protein A, the protein A concentration approximates 13 mg/mL. For a bead packing efficiency of 0.75, there are approximately 6.6 million beads in 1 mL of resin. Estimating the resin surface area brings a more considerable increase in error. According to Harrison et al. [47] the ratio of bead outer surface area to inner pore area is between 1:60 and 1:800. Thus, if the outer surface area of 6.6 million 60 μm beads equals 0.075 m^2^, the inner pore area could be between 4.5 and 60 m^2^. Therefore, we estimate the protein A surface coverage in Prosep Ultra Plus to be between 0.2 and 2.9 mg m^−2^. We note that several assumptions have been made in order to arrive at these figures. What we can infer from this comparison is that the protein A coverage estimated in this work is within the expected order of magnitude, and towards the low end.

The addition of BSA caused small shifts in SLD values, indicating that a small quantity of BSA was adsorbed in the blocking process. The volume fraction estimate for the protein layer increased by about 1.5% after BSA addition. This is in agreement with the FTIR data (Fig. 4). The decrease in SLD in the D_2_O contrast after BSA blocking extended across the full length of the protein A layer, suggesting that BSA retained a folded structure and extended out into the solution phase.

#### 4.5.2 IgG adsorbed to protein A/BSA surface

**Fig. 6:**
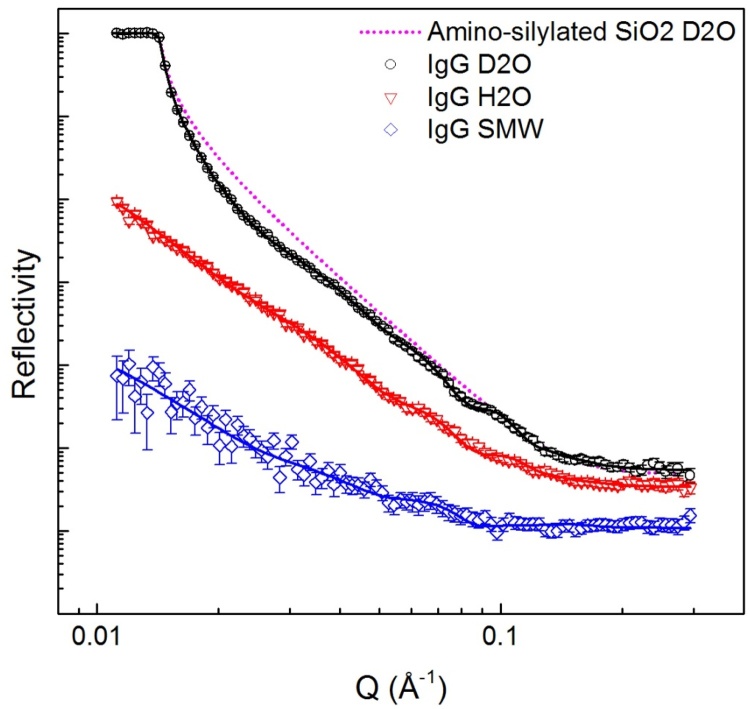
Reflectivity (intensity) against Q at three solution phase contrasts for IgG4 adsorbed at pH 6.7 to rSPA-modified silica with BSA blocking. Black, red and blue data points represent D_2_O, H_2_O and silicon matched water (SMW, 38% D_2_O) solution phases, respectively. H_2_O and SMW data sets are offset down the y-axis to prevent overlap. Solid lines represent fitted models. The pink dotted line represents the fitted model for the amino-silylated surface in D_2_O (Fig. 5) and is shown for comparison. (For interpretation of the references to colour in this figure legend, the reader is referred to the web version of this article.)

When IgG was adsorbed in cell A there was sufficient interfacial structure to fit SLD-distance profiles using all three solution-phase contrasts; thus, there was a high degree of confidence in the model. Looking at the data for D_2_O-based conditions in Fig. 6, a broad fringe can be seen covering Q values 0.014–0.04 Å^−1^; fringes in the low Q region arise due to large structures that extend away from the interface. Another clear fringe is around 0.08 Å^−1^. Importantly, the shape of this reflectivity profile is very different to those seen in Fig. 2, Fig. 3, indicating that non-specific adsorption to the surface did not take place.

**Fig. 7:**
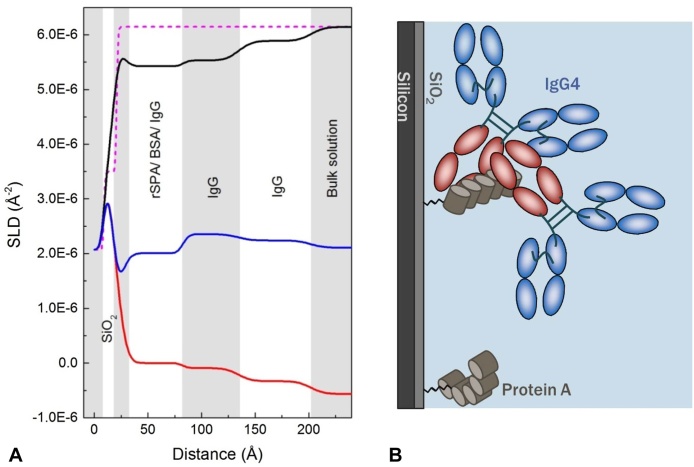
(A) Profile of scattering length density against distance for IgG4 adsorbed at pH 6.7 to protein A-modified silica with BSA blocking. Data was fitted simultaneously for three solution phase contrasts: D_2_O (black), H_2_O (red) and *silicon matched water* (SMW) (blue). The pink dotted line represents the bare amino-silylated silicon wafer in D_2_O. Shading is to aid layer visualisation. Layer identities are suggested in black text. (B) Schematic of one suggested configuration for IgG4 adsorbed to protein A on a silicon substrate; BSA has been omitted for simplicity. (For interpretation of the references to colour in this figure legend, the reader is referred to the web version of this article.)

The best fit for the data was achieved using a 5 layer model (including the SiO_2_ layer). The resulting SLD profiles are shown in Fig. 7. The profile suggests three IgG-related layers with a total thickness of 180 Å. The innermost of these layers overlaps with the protein A/BSA layer that was defined before IgG addition (Fig. 5); there is also some overlap between this and the middle IgG-related layer. The inner, middle and outer IgG-containing layers had thicknesses of 61 Å, 52 Å and 67 Å, respectively. Layer volume fractions were estimated using Eq. (4); the inputs and outputs of these calculations are displayed in Table 3. The innermost IgG-related layer had a volume fraction of 0.25; thus, IgG made a contribution of 0.18 to the layer volume fraction – 3.6 times the contribution from protein A and 2.6 times that from protein A and BSA combined (Table 3). The ratio of IgG to protein A is similar to that seen in FTIR measurements; however, in this case we are looking at only the innermost IgG-related layer where two additional layers are present. Assuming overlap between protein A and IgG in the middle IgG-related layer, IgG made a contribution of 0.14–0.16 to the (middle) layer volume fraction. In the outermost layer, which was assumed to have arisen from IgG structure only, the volume fraction was lower at 0.09. The total depth of the IgG-related layers was greater than the maximum length of a single IgG4 molecule, 160 Å [4]. Therefore, we suggest that the three-layer structure was a result of two IgG molecules bound to protein A in side-on or skewed orientations. If we consider the 6-domain structure of rSPA (including the X_t_ domain), the fully extended molecule could have a length of more than 130 Å [14]. We suggest that when IgG binds it supports the flexible protein A structure causing it to adopt a more extended conformation, and resulting in a volume fraction contribution from protein A along a greater distance in the z direction (i.e. in the first *and* second IgG-related layers). One IgG may bind to one side of protein A; a second IgG molecule may then bind to a more surface-distal domain of protein A. One example of this type of arrangement is illustrated as a cartoon in Fig. 7b. The outermost layer could be the result of a single Fab arm extending into the solution, as its length is representative of an IgG4 Fab arm [28]. To give another example, rather than the total IgG depth being the result of two IgG molecules bound to each protein A ligand, it could be that one IgG was bound to a protein A domain close to the solid-liquid interface, in a side-on to tilted orientation, while another IgG was bound to an adjacent protein A molecule at a domain further from the interface. There are a number of possible arrangements. It is difficult to draw firm conclusions about the orientation of adsorbed IgG without isotopic labelling of at least one of the protein components. We note that the bootstrap error analysis indicated the SLDs of the IgG-related layers to be highly robust. Therefore, despite uncertainty regarding interpretation of the model, the model itself can be considered an accurate representation of the interfacial macro-structure for the given system. A related consideration is that IgG has been shown to bind two protein A ligands simultaneously [3]. Due to the relatively sparse surface coverage of protein A, we suggest that this was not the case. Further, near-complete elution of the IgG at pH 4.1 suggests that IgG molecules were “single site bound” [3].

#### 4.5.3 Protein A/PEG_6000_ surface characterisation

**Fig. 8:**
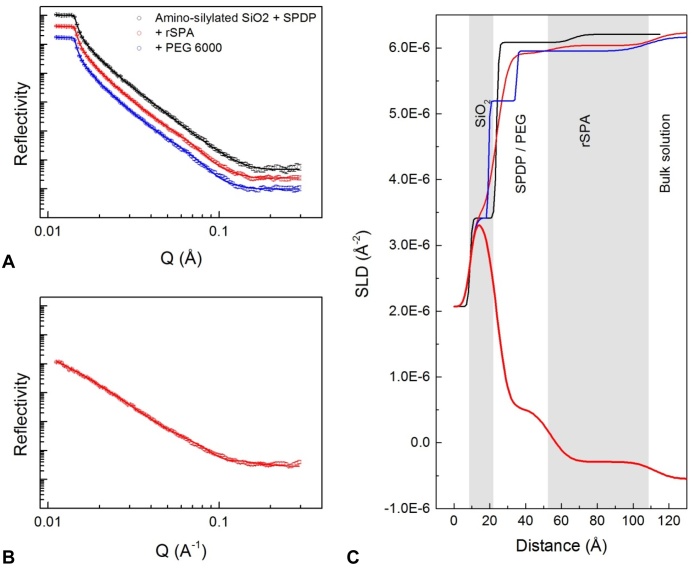
Reflectivity data for attachment of rSPA to an amino-silylated silica surface, and addition of the blocking agent PEG_6000_. Left-hand plots A (D_2_O contrast) and B (H_2_O contrast): black is the amino-silylated wafer with cross-linker attached; red is after rSPA cross-linking and blue is after blocking with PEG_6000_. Solid lines show the fitted models. Plot C shows the corresponding profiles of scattering length density, SLD (Å^−2^), against distance from the silicon surface. Where data was collected at both solution phase contrasts, the profile was generated by simultaneous fitting of D_2_O and H_2_O reflectivity data sets to a single layer depth profile. (For interpretation of the references to colour in this figure legend, the reader is referred to the web version of this article.)

In the second sample cell (B, Table 2) rSPA was cross-linked to the surface in the same way as before, but PEG_6000_ was used as a blocking agent instead of BSA (Table 2); the related reflectivity data is shown in Fig. 8. The layer structure after rSPA cross-linking is similar to that seen in cell A. The data suggests a slightly higher coverage of protein A (Table 4) and a near-identical thickness for combined cross-linker and protein A layers. The addition of PEG caused a reduction in the SLD of the innermost layer (after SiO_2_), though data was collected for the D_2_O contrast only (Fig. 8). This reduction in SLD is expected because PEG has few exchangeable hydrogens. The fit error analysis showed that the SLD and thickness parameters were robust for the protein A layer (after PEG blocking), but less-so for the PEG/cross-linker layer.

#### 4.5.4 IgG adsorbed to protein A/PEG_6000_ surface

**Fig. 9:**
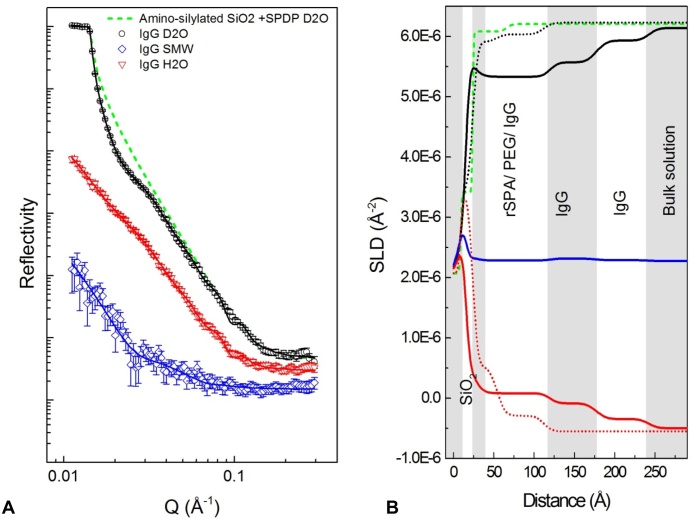
Reflectivity data for IgG4 adsorbed at pH 6.7 to rSPA-modified silica with PEG_6000_ blocking. Left-hand plot (A): reflectivity (intensity) against momentum transfer, Q (Å^−1^), at three solution phase contrasts: D_2_O (black), H_2_O (red) and silicon-matched water (blue). H_2_O and SMW data sets are offset down the y-axis to prevent overlap. The fitted curve for the amino-silylated surface with cross-linker only is shown for comparison (green dotted line). Plot B shows the corresponding profiles of scattering length density (Å^−2^) against distance from the silicon surface. Black, red and blue curves represent D_2_O, H_2_O and SMW data, respectively. SLD-distance profiles for the surface with cross-linked rSPA (dotted lines) and with cross-linker only (green dashed line) are shown for comparison. (For interpretation of the references to colour in this figure legend, the reader is referred to the web version of this article.)

The profile generated after IgG adsorption in cell B (Table 2 and Fig. 9) shares a number of key characteristics with that seen in cell A (Table 2 and Fig. 7), such as three major IgG-related layers; there are also some differences. The total depth of cross-linker/rSPA layers was highly similar in cells A and B before IgG adsorption. However, when IgG was adsorbed the innermost IgG-related layer had a significantly greater depth in cell B compared to cell A; the SLD in the D_2_O contrast was also slightly lower (Table 4). The outer two layers were comparable in thickness and SLD values across the two cells for the D_2_O and H_2_O contrasts, but not for the SMW contrast; here, little structure was resolved at the solid-liquid interface. This is not the result of similar SLD values for pure protein and silicon. We note that the fit to the data is not perfect in the D_2_O contrast. Specifically, there is a fringe in the fitted curve, at a Q value just below 0.1 Å^−1^, that falls a little outside the data points (Fig. 9c). It was not possible to eliminate this discrepancy with the computational resources available. In the H_2_O and SMW contrasts the fits to the data points were satisfactory; thus, the overall fit was accepted.

**Fig. 10:**
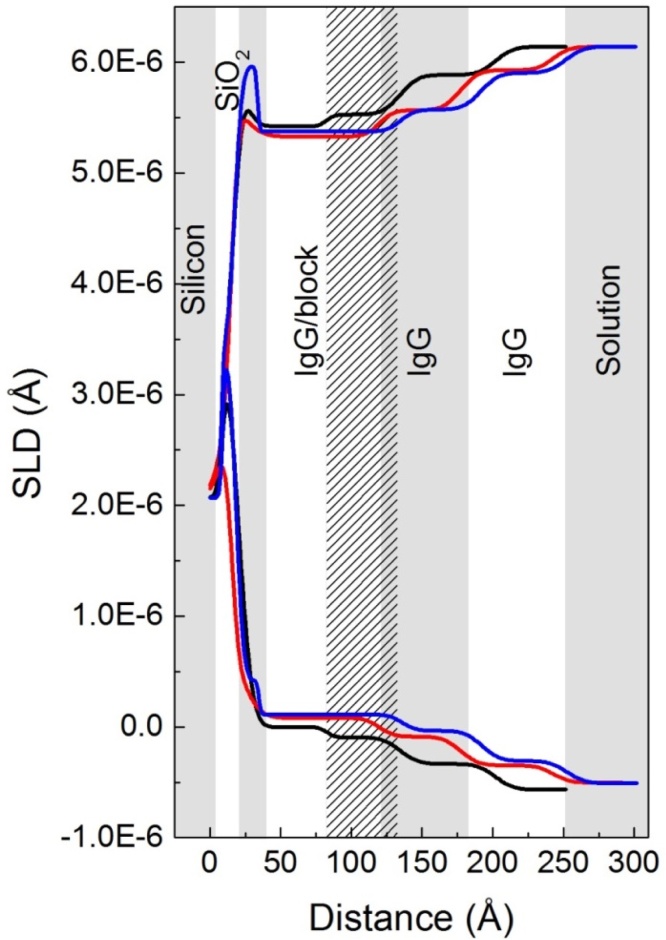
SLD-Distance profiles for IgG adsorbed to protein A with BSA surface blocking (black line) and PEG_6000_ surface blocking in Cell B (red line) and Cell C (blue line). (For interpretation of the references to colour in this figure legend, the reader is referred to the web version of this article.)

In cell C (Table 2) the surface was generated in the same way as in cell B, and when IgG4 was adsorbed the fitted models were very similar in both cells. This can be seen in Fig. 10 where the SLD-distance profiles for the three sample cells are compared in the D_2_O and H_2_O contrasts. Fitted SLDs and calculated volume fractions are summarised in Table 4. Comparing the volume fraction values in Table 4 to those in Table 3, we note a slight shift in distribution of the layers (when IgG is adsorbed), with volume fractions slightly higher for the inner IgG-related layer and slightly lower for the middle layer in cells B and C. However, these differences are slight – the main difference is in the thickness of the inner IgG-related layer. The data suggests that using PEG as a blocking molecule instead of BSA had a significant effect on the orientation of adsorbed structures at the solid-liquid interface. The surface-proximal IgG molecule may adopt a more upright orientation, possibly due to repulsive effects of PEG. Subsequently, the second IgG molecule becomes more distal from the surface, extending up to 250 Å away from it. This theory shares some similarity with data obtained by Le Brun et al. [48], in which addition of thioPEG was found to “fill out” space in a layer of protein covalently bound to a gold surface. The protein in this case was termed *ZZctOmpA* and consisted of a beta barrel domain and two protein A *Z* domains (for a description of the Z domain, see Lain [46]). The system shared similarities with that discussed in this work, including use of neutron reflection methods. Indeed, the SLD of the ZZctOmpA layer was similar to that of the rSPA layer in this work, and after antibody addition a total layer depth of around 200 Å and an SLD of approximately 5.5 × 10^−6^ Å^−2^ was obtained for the antibody/ZZctOmpA layer [48]. The data of Le Brun et al. was modelled such that the antibody region was well-fitted as a single layer, and interpreted as a 1:1 ratio of ZZctOmpA to antibody, with the antibody in an “upright” orientation [48]. This differs to the data presented here, in which the best fit models showed three IgG-related layers, suggestive of skewed IgG orientation and a higher ratio of IgG to protein A. We note that in this work up to 5 IgG-binding domains were available, while in that of Le Brun et al. [48] there were only two.

## 5 Conclusions

In the first part of the work, we showed that IgG4 adsorbs directly to silica at a high packing density in a largely flat-on orientation at pH 4.1. While the IgG solution remained in the cell a fraction of weakly adsorbed material was also observed; data suggested partial re-orientation of this material into the flat on-configuration on rinsing the surface with buffer. Altering the pH affected the orientation and packing density of adsorbed IgG. The data demonstrated that electrostatic attraction between IgG and surface was a strong driving force for surface adsorption, and protein-protein repulsion was a factor that influenced the packing density of the interfacial layer. In the second part of the work a model surface including recombinant staphylococcal protein A cross-linked to silica, and one of two blocking molecules, BSA or PEG_6000_, was examined by FTIR and neutron reflectivity. The FTIR data indicated that rSPA was successfully cross-linked to the surface and retained its structure for IgG binding. IgG was found to elute at a higher pH than is typically expected. FTIR data also demonstrated a lack of non-specific binding to the model surface. These findings were corroborated by reflectivity data. Estimates of rSPA surface coverage were compared to approximations of protein A coverage on the porous glass resin Prosep (Millipore); it was inferred that surface coverage estimates for rSPA were within the expected range, and towards the low end. Apparently low protein A surface coverage coupled with IgG elution at pH 4.1 suggested that each *IgG* was bound to only one protein A, supporting a “single/dual site binding” theory described by Gagnon and Nian [3]. Analysis of the reflectivity data revealed layer-based model structures in which it was possible to identify distinct contributions from protein A and from IgG4. It was clear that IgG was adsorbed to protein A, and not non-specifically adsorbed to the surface. Key structural features included three protein layers extending to a maximum of 250 Å away from the solid surface into the solution. Layer volume fractions showed robust fits for all three sample cells used, suggesting that the IgG orientation was specific and consistent. The data indicated that the different blocking molecules influenced the IgG layer structure, with the use of the PEG_6000_ as a block resulting in more extended structures. Though the data was reproducible and data fits largely robust, it was difficult to interpret the exact macro-structures based on the data sets acquired. Nonetheless, the data gave a new insight into the arrangement of IgG adsorbed to immobilised protein A in terms of the distribution of protein layers in the surface normal direction, and their respective volume fractions. The influence of other surface molecules on the orientation of the IgG and its interaction with protein A was also highlighted. Finally, we acknowledge that the flat, polished silica surface used for reflectivity experiments is a very different physical configuration to typical porous chromatography resins. We propose the use of small angle neutron scattering to probe these protein structures in porous silica beads, which would be a true representation of the system of study. Scattering from the beads can be eliminated by contrast-matching to the solution phase, revealing the structure of the proteins within.

## Figures and Tables

**Table 1:** Model outputs for IgG4 adsorption to silica at pH 4.1. Theoretical SLD values for IgG4 at 0% and 90% H-D exchange levels; scattering length densities of the mobile phases and the inner and outer protein layers from fitted models; estimated protein volume fractions for each layer, and chi squared vales for the two fits.

**Table 2:** Chi-squared values for fitted models for different sample configurations across the three solid-liquid flow cells (A–C). Models were fitted using data sets from one, two or three solution phase contrasts, indicated by shading.

**Table 3:** Volume fraction estimates for layers of various sample configurations in cell A. Volume fraction estimates were made based on the theoretical SLD values for the pure proteins at 90% H-D exchange. SLDs for pure rSPA and IgG were calculated using the Biomolecular Scattering Length Density calculator at http://psldc.isis.rl.ac.uk/Psldc/. Volume fraction estimates were calculated using Eq. (4).

**Table 4:** Volume fraction estimates for layers of various sample configurations in cells B and C. See Table 3 for calculation details.
