# Supplementary material for: Neutron reflectivity measurement of protein A–antibody complex at the solid-liquid interface
Source: J Chromatogr A. 2017 May 26;1499:118–31. doi: 10.1016/j.chroma.2017.03.084 (PMC5408906; doi:10.1016/j.chroma.2017.03.084)
Supplement: Supplementary file 1 [file mmc1.docx]

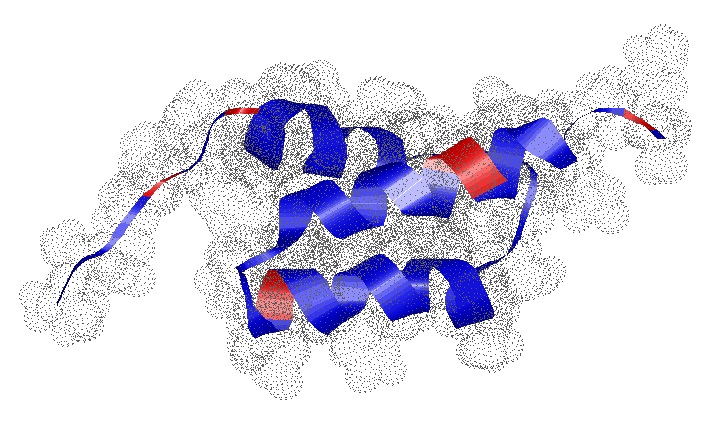

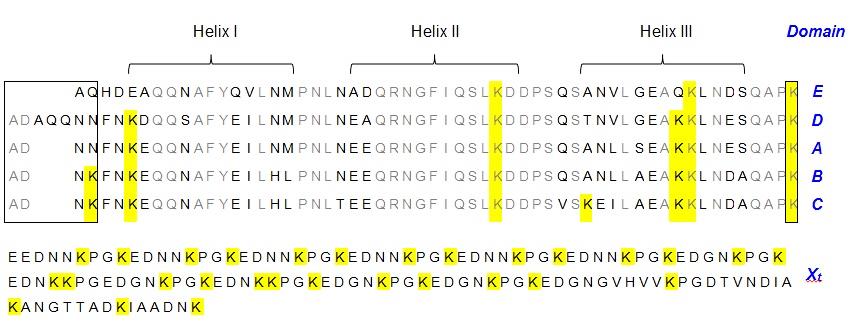


**B**

**A**

Supplementary Figure 1 (A) NMR structure of the B domain of protein A; positions of lysine residues are highlighted in red. NMR structure PDB ID 1BDD, (B) Amino acid sequence of recombinant staphylococcal protein A (rSPA) from Repligen. E, D, A, B and C domains are aligned to show sequence homogeneity. Residues that are identical across domains are shown in grey, those that vary across domains are in black and lysine residues are highlighted in yellow. Unstructured regions that link each three-helix bundle are within black boxes. The truncated X domain is denoted Xt and is shown as a continuous string of residues in black.

The NMR structure of domain B of protein A was originally published by Gouda et al. (1992) [49]. The visual representation of the structure was edited using RasMol software [50, 51]. The sequence for recombinant staphylococcal protein A is from Peyser (2010) [33] and the sequence layout was adapted from Capp et al. (2014) [14].
